# Endothelial Secreted Factors Suppress Mitogen Deprivation-Induced Autophagy and Apoptosis in Glioblastoma Stem-Like Cells

**DOI:** 10.1371/journal.pone.0093505

**Published:** 2014-03-28

**Authors:** Eva Maria Galan-Moya, Lucas Treps, Lisa Oliver, Hervé Chneiweiss, François M. Vallette, Nicolas Bidère, Julie Gavard

**Affiliations:** 1 UMR 8104, Centre National pour la Recherche Scientifique, Paris, France; 2 U1016, Institut National de la Sante Et de la Recherche Medicale, Paris, France; 3 Sorbonne Paris Cite, Universite Paris Descartes, Paris, France; 4 UMR 892, Institut National de la Sante Et de la Recherche Medicale, Nantes, France; 5 UMR 6299, Centre National pour la Recherche Scientifique, Nantes, France; 6 Faculte de Medecine, Universite de Nantes, Nantes, France; 7 Institut de Cancerologie de l'Ouest, Nantes, France; 8 Equipe Labellisee Ligue contre le Cancer, Paris, France; 9 UMRS 1130 Neuroscience Paris Seine, Institut National de la Sante Et de la Recherche Medicale, Paris, France; 10 UMR 8246, Centre National pour la Recherche Scientifique, Paris, France; 11 UMCR18, Université Pierre et Marie Curie, Paris, France; 12 UMR_S1014, Institut National de la Sante Et de la Recherche Medicale, Villejuif, France; 13 Universite Paris-Sud P11, Orsay, France; University Dresden, Germany

## Abstract

Rapidly growing and highly vascularized tumors, such as glioblastoma multiforme, contain heterogeneous areas within the tumor mass, some of which are inefficiently supplied with nutrients and oxygen. While the cell death rate is elevated in such zones, tumor cells are still suspected to grow and survive independently of extracellular growth factors. In line with this, glioblastoma stem-like cells (GSCs) are found closely associated with brain vasculature *in situ*, and as such are most likely in a protected microenvironment. However, the behavior of GSCs under deprived conditions has not been explored in detail. Using a panel of 14 patient-derived GSCs, we report that *ex vivo* mitogen deprivation impaired self-renewal capability, abolished constitutive activation of the mTor pathway, and impinged on GSC survival via the engagement of autophagic and apoptotic cascades. Moreover, pharmacological inhibition of the mTor pathway recapitulated the mitogen deprivation scenario. In contrast, blocking either apoptosis or autophagy, or culturing GSCs with endothelial-secreted factors partly restored mTor pathway activation and rescued GSC survival. Overall, our data suggest that GSCs are addicted to mTor, as their survival and self-renewal are profoundly dependent on this signaling axis. Thus, as mTor governs the fate of GSCs under both deprivation conditions and in the presence of endothelial factors, it could be a key target for therapeutic purposes.

## Introduction

Adult glioblastoma multiforme (GBM) is the most common primary brain tumor. Patients with malignant glioma have poor prognosis due to local recurrence. Despite surgery, radiotherapy and temozolomide chemotherapy, less than 10% of patients reach a 5-year survival [1]. GBMs comprise a subpopulation of cells, which retain the ability to expand *ex vivo* as neurospheres in mitogen-defined medium and recapitulate tumor formation in mice [2], [3]. These so-called glioblastoma stem-like cells (GSCs) share many characteristics with normal stem cells, including self-renewal and multipotency [3]. One major mechanism that eliminates undesired and potentially toxic cells is apoptosis [4]. However, whether apoptosis contributes to limit stem cell expansion has not been completely elucidated. For instance, we have shown that adult human mesenchymal stem cells do not undergo apoptosis when left undifferentiated [5]. Moreover, the impact of apoptosis on stem cells and cancer stem-like cells during tissue regeneration and tumor progression is not known. The manipulation of autophagy has also been suggested as a valuable strategy to prevent cancer development, limit tumor progression and increase the efficacy of cancer treatments [6]. Therefore, while GBMs are generally resistant to therapies that induce apoptosis, they might be more sensitive to those targeting autophagy [7]. However, the molecular mechanisms governing the interplay between autophagy and apoptotic cell death in GSCs remain to be elucidated.

Although neo-angiogenesis is believed to overcome growing tumor needs, inner zones within the tumor mass are not always efficiently irrigated and an increase in the tumor cell death rate can occur as a consequence of starved conditions [8]. Likewise, penury can be provoked upon administration of chemotherapeutic and anti-angiogenic agents. Indeed, GSCs can be suppressed along with tumor endothelial cells in response to anti-vascular endothelial growth factor antibody challenge [9]. Here, we show that mitogen deprivation induces both autophagy and apoptosis in patient-derived GSCs. This effect is largely dependent on mammalian target of rapamycin (mTOR) activation and can be alleviated by the endothelial cell secretome. Moreover, blockade of either autophagy or apoptosis can restore the activation of mTor. In agreement with its role in sensing microenvironmental conditions, mTor could thus chiefly orchestrate the fate of the GSC population.

## Materials and Methods

### Cell culture

Patient-derived GSCs #1 to 4 were described previously in [10]–[13], GSCs #5 to 14 were obtained from Hospital Laennec, Nantes, France. Briefly, tumors were dissociated using the gentleMACs Dissociator (Miltenyi), according to the manufacturer’s instructions. GSCs #1 to 14 were maintained in DMEM/F12 plus N2, G5 and B27 (Life Technologies). These three supplements were omitted when cells were cultured under mitogen deprivation. Differentiated GSCs were obtained by dissociation and cultured in DMEM plus 10% FBS and 1% penicillin/streptomycin (Life Technologies). Concomitant increase in the differentiation marker GFAP and decrease in the stemness markers Nestin and Sox2 were observed after 3 days of adhesion. Immortalized human brain microvascular endothelial cells (hCMEC/D3) were expanded as described previously [11]–[14]. Conditioned media from hCMEC/D3 (EC-CM) were obtained from 72h-old monolayers cultured in serum-free EBM2 (Lonza), as previously described [11].

### Ethics statement

Patients all signed a written informed consent before sample collection, which occurred for diagnostic purposes. This study was reviewed and approved by an institutional review board (Sainte Anne Hospital, Paris, France, and Laennec Hospital, Nantes, France). This study abides the rules of the Helsinki Protocol.

### Reagents and antibodies

Rapamycin and PI103 were purchased from MERCK, LY294002 from Tocris, PP242 from Sigma, quinolyl-valyl-O-methylaspartyl-[–2,6-difluorophenoxy]-methyl ketone (QVD) was from R&D systems. The following antibodies were used: phospho (p)S-Akt, pT-Akt, p-S6, p-4EBP1, pAMPK, pACC, LC3B, Atg12, Bim, Caspase 3, and Bcl-2 (Cell Signaling Technology), Cytochrome-c and Tubulin (Santa Cruz), Sox2 (Millipore), and GFAP (Sigma). Stealth non-silencing control and selected siRNA for human Beclin were from Life Technologies. Transfections were performed using RNAiMAX lipofectamine (Life Technologies). Alexa488- and Alexa546-conjugated antibodies were purchased from Life Technologies.

### Secondary neurosphere formation

GSCs were dissociated by up-and-down pipetting and cultured in a 48-well plate format. Cells were dissociated again at days 1 and 2 after mitogen deprivation and then maintained until day 3. Neurosphere (NS) counts were blindly performed on five random fields of view (fov) and the mean of NS/fov was calculated from 3 independent experiments. Statistical analyses were performed using two-way Student’s test.

### RT-PCR

RNA was extracted using the RNeasy Mini Kit as per the manufacturer’s directions (Qiagen). Equal amounts of RNA were reverse transcribed using the Maxima Reverse Transcriptase (Thermo Scientific) and the resulting cDNA was used to amplify Nestin and Sox2 transcripts by PCR using gene-specific primers for human Sox2 (Fwd: 5′-CAAAAATGGCCATGCAGGTT-3′; Rev: 5′-AGTTGGGATCGAACAAAAGCTATT-3′) and human Nestin (Fwd: 5′-TTCTCTTGTCCCGCAGACTT-3′; Rev: 5′ AACAGCGACGGAGGTCTCTA-3′) in the presence of RedTaq Mix (Sigma), as described previously [15]. Human GAPDH (Fwd: 5′-ACTTCACCTTCCCTCCAACC-3′; Rev: 5′-GGAGGAGTGGGTGTCGCTGT-3′) was also amplified as a control for input. PCR products were separated by electrophoresis on SYBR safe-containing agarose gels (Life Technologies).

### Western blot

Proteins were collected in TNT buffer (10 mM Tris-HCl pH 7.5, 150 mM NaCl, 1% Triton X100, 2 mM EDTA) plus protease inhibitors (Sigma), 200 mM NaF and 0.1 mM Na3VO4. Equal amounts of proteins (microBCA kit, Pierce) were separated with 4–12% Nupage gels (Life Technologies) and transferred onto PVDF membranes (Thermo Fisher Scientific). Alexa680-conjugated secondary antibodies (Life Technologies) were used and membranes were scanned using the Odyssey infrared imaging system (Licor).

### Confocal and electron microscopy

Cells were dropped on poly-lysine-coated slides (Thermo Fisher Scientific) and fixed in PBS-paraformaldehyde 4%, either directly or after previous mechanical dissociation. Immunofluorescence was performed as previously described [11], using Alexa488-conjugated secondary antibodies (Life Technologies). Samples were mounted in DAPI-containing mounting medium (Prolong gold antifade reagent, Life Technologies). Confocal acquisitions were performed on TCS/SP4 Leica confocal microscope (Institut A. Lwoff, Villejuif).

Following the indicated treatments, cells were collected and fixed for electron microscopy (3% glutaraldehyde/PBS, 1 hour). After incubation in 1% OsO4, cells were dehydrated in graded dilutions of ethanol, embedded in artificial resin (Epon, Momentive Specialty Chemicals) and processed for electron microscopy. GSC morphology was monitored by transmission electron microscopy (EM10CR, Zeiss, Imagery facility, Institut Cochin) performed at 60–80 kV on unstained thin sections.

### Flow cytometry

Apoptosis was evaluated using both the FITC Annexin V/Dead Cell Apoptosis Kit as per the manufacturer’s instructions (Life Technologies) and sub-G1/G0 population quantification. For the latter, cells were fixed with cold ethanol 70% for 15 min, wash twice with cold PBS, and RNase (100 μg/ml) and propidium iodide (PI, 40 μg/ml, Calbiochem) were added to the samples 10 min prior analysis. Cell death was determined by PI staining (50 μg/ml). Mean survival (PI exclusion) was calculated from 3 independent experiments. Statistical analyses were performed using two-way Student’s test. All flow cytometry analyses were performed on Accuri cytometer (BD Biosciences, CYBIO facility, Institut Cochin) and processed using CFlow plus software (BD Biosciences).

## Results

### Mitogen deprivation reduces GSC survival

**Figure 1:**
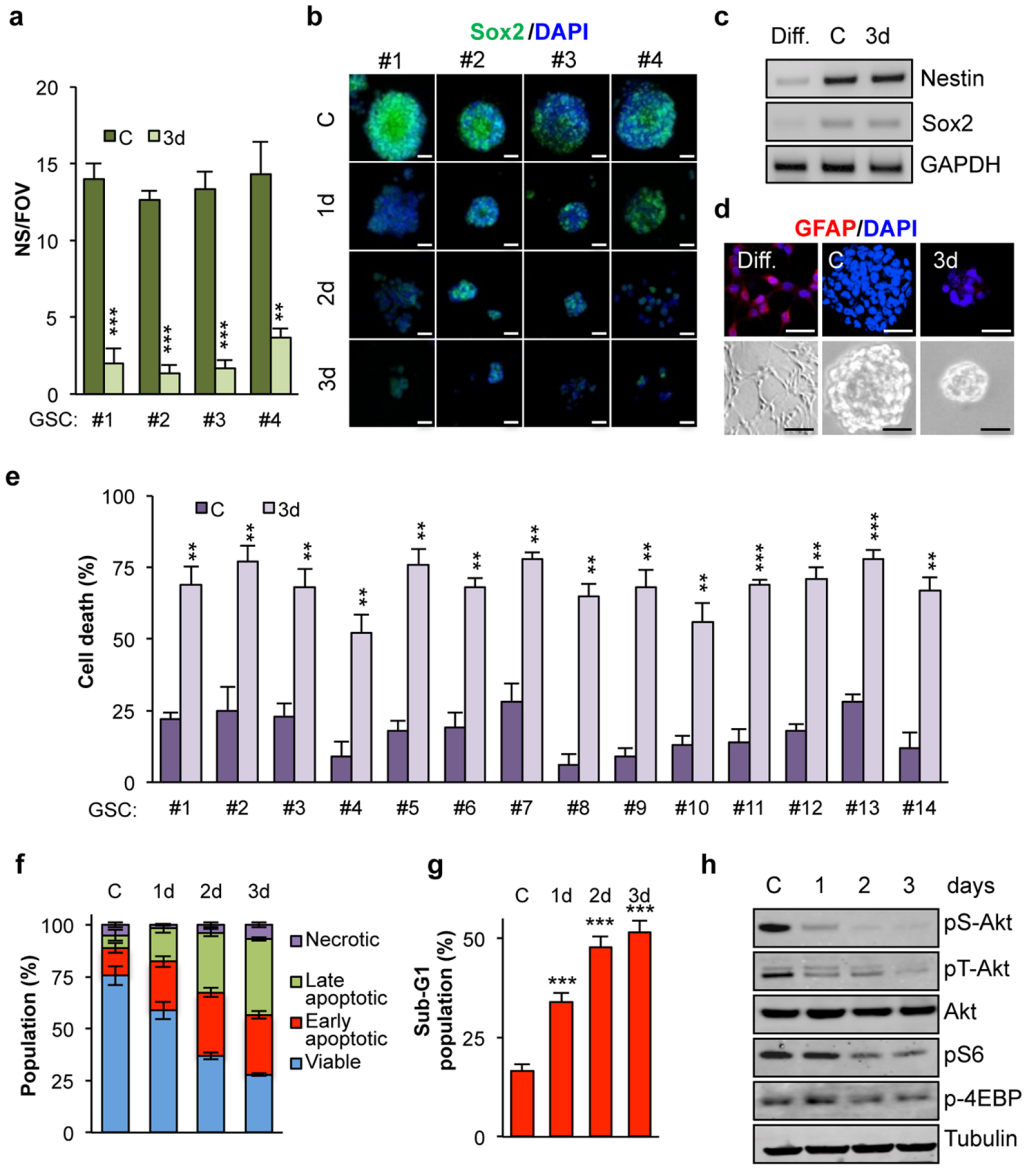
Mitogen deprivation reduced glioblastoma stem-like cell survival. a-h) GSCs were deprived from mitogens for 1 to 3 days. GSCs growing in mitogen-supplemented medium were used as a control (C). a) The number of secondary neurospheres per field of view (NS/FOV) was counted after 3 days of deprivation in GSCs #1-4. b) Sox2 levels (green) were analyzed by confocal in GSCs #1-4. Nuclei were counterstained with DAPI (blue). Scale bar: 15 μm. c-d) Expression of Sox2 and Nestin as well as GADPH as a control, was evaluated by RT-PCR, while GFAP expression was analyzed by confocal analysis. Differentiation of GSC#4 was induced as described in methods. e) PI incorporation was measured by flow cytometry (10.000 events) in GSCs #1–14 and the percentage of cell death was estimated. Graph represents mean+s.d. of three independent experiments. Student’s t-test: ^***^ *P*<0.001, ^**^ *P*<0.01. f) Flow cytometry analysis (10.000 events) of Annexin V-FITC/Propidium Iodide (PI) staining was used to measure cell death in GSC#1. Number of cells either viable (Annexin V/PI –/–) or in early (Annexin V/PI +/–), late apoptosis (Annexin V/PI +/+) and necrotic (Annexin V/PI –/+) phases was expressed as percentage of total population. g) DNA profile was analyzed by flow cytometry with PI staining in GSC#1. Percentage of apoptotic cells was calculated based on sub-G_1_ peak. Student’s t-test: ^***^ *P*<0.001. h) Western blot analysis was conducted using the indicated antibodies in GSC#1. Each panel is representative of three independent experiments.

Fourteen patient-derived GSCs, which efficiently form neurospheres (NS) and express stem cell markers, such as the transcription factor Sox2, among others, were maintained *ex vivo* in a serum-free medium containing a defined cocktail of mitogens [10]–[13]. To study GSC behavior under deprivation conditions, which could occur within the tumor mass, we first investigated self-renewal ability in the absence of mitogens. Mitogen deprivation dissipates self-renewal hallmarks, such as secondary NS formation and nuclear expression of Sox2 (Fig. 1a-b), suggesting that deprivation conditions lead to loss of GSC identity. We then explored whether these culture conditions could promote GSC differentiation, as changes in mitogen concentration have been shown to contribute to differentiation of stem cells [16]. However, the expression of the stemness markers, Sox2 and Nestin, was not altered, at the RNA level, by mitogen withdrawal in non-adhesive cultures, as compared to adherent and morphologically differentiated GSCs (Fig. 1c-d). Meanwhile the expression of the astrocyte intermediate filament protein, glial fibrillary acidic protein (GFAP) was clearly turned on in adherent cells, while barely detectable in either growing or mitogen-deprived GSCs (Fig. 1d). This suggests that deprivation does not induce differentiation. Alternatively, the failure in NS formation under mitogen-deprived conditions could be due to either a reduction in GSC survival or the establishment of a quiescent non-proliferative state [17]. Of note, cell death was increased in a panel of fourteen patient-derived GSCs cultured under mitogen deprivation for three days (Fig. 1e). Flow cytometry analysis of Annexin V/propidium iodide (PI) staining indicated that mitogen deprivation indeed triggered apoptotic cell death, as survival dropped from 75% to less than 30% after three days (Fig. 1f). Analysis of nuclear DNA content further showed that more than 50% of three days-starved GSCs exhibited a sub-G1/G0 phase profile, typical of apoptosis-related DNA fragmentation (Fig. 1g). We next investigated the status of the mTor (mammalian target of rapamycin) pathway, a crucial signaling nexus for GSC integrity [18]. Notably, the phosphorylation of key mediators of this molecular cascade, namely Akt, S6 and 4EBP, was compromised as early as 24 hours post-starvation, suggesting that mitogen additives sustain the constitutive activation of the mTor pathway in GSCs (Fig. 1h). Together, this set of experiments suggests that mitogen deprivation drives cell death in GSCs, together with a decrease in mTor activation.

### Mitogen deprivation triggers both autophagy and apoptosis in GSCs

**Figure 2:**
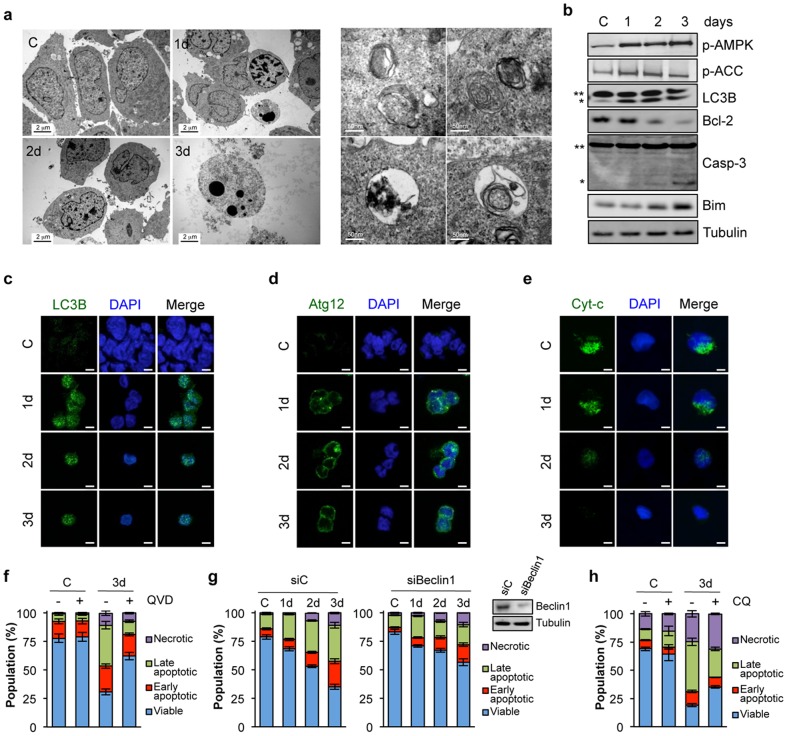
Mitogen deprivation triggers both autophagy and apoptosis. a-e) GSCs were deprived from mitogens for 1 to 3 days. GSCs growing in mitogen-supplemented medium were use as a control (C). a) Electron microscopy analysis of mitogen-starved GSC#1. *Left panels*. Cells presented morphological signs of apoptosis, including cytoplasmic blebbing, nuclear fragmentation and chromatin condensation. Scale bars: 2 μm. *Right panels*. Different stages of autophagy can be seen, such as pre-autophagosome and early autophagosome (upper panels) and late autophagosomes (autolysosomes/amphisomes, lower panels). Scale bars: 50 nm. b) Western blot analysis was conducted using the indicated antibodies in GSC#1. ** non-processed form; * processed form. c-e) LC3B puncta, Atg12 aggregates and mitochondrial cytochrome c release were analyzed by confocal microscopy. Scale bars: 5 μm (c,d) and 2.5 μm (e). f) GSC#1 were pre-treated with vehicle (DMSO) or QVD (10 μM) for 45 min and mitogen-deprived for 3 days in the absence or presence of the drug. Flow cytometry analysis (10.000 events) of Annexin V-FITC/PI staining was used to measure cell death in GSC#1. Number of cells either viable (Annexin V/PI –/–) or in early (Annexin V/PI +/–), late apoptosis (Annexin V/PI +/+) and necrotic (Annexin V/PI –/+) phases was expressed as percentage of total population. g) GSC#1 were transfected with siRNA against Beclin-1 or a control siRNA (siC) for 48 hours. Cells were mitogen-deprived as indicated and collected 5 days post-transfection. Beclin-1 protein levels were analyzed by western-blot and cell death was examined as in f). h) GSC#1 were treated with vehicle (DMSO) or Chloroquine (CQ) (25 μM, XX min) in mitogen-supplemented medium (C) and in mitogen-deprived conditions for 3 days (3d). Flow cytometry analysis (10.000 events) of Annexin V-FITC/PI staining was used to measure cell death in GSC#1 as in f). Graph represents mean+s.d. of three independent experiments. Each panel is representative of three independent experiments.

To better characterize how mitogen deprivation promoted cell demise, GSC morphology was evaluated using electron microscopy (Fig. 2a). In deprived cells, both autophagic and apoptotic signatures were clearly identified, such as double membrane organelles and nuclear condensation, respectively. Moreover, deprivation triggered the phosphorylation of key sensors of autophagy [19], [20], ie AMPK (AMP-activated protein kinase) and its downstream target ACC (acetyl-coA carboxylase) (Fig. 2b). Further confirming autophagosome formation, LC3B lipidation and punctuation, and ATG12 aggregation, were increased (Fig. 2b-d) [21]–[23]. Paralleling autophagy induction, starvation also induced dissipation of mitochondrial cytochrome c and cleavage of the effector caspase-3, indicative of caspase-dependent apoptosis (Fig. 2b, 2e). In agreement with these data, the expression of the pro-survival Bcl2 protein was decreased over time, while that of the pro-apoptotic BH3-only protein Bim gradually increased (Fig. 2b) [24]. Treatment with the broad-spectrum caspase inhibitor QVD [25] minimized deprivation-induced GSC apoptosis and partly restored cell survival (Fig. 2f). To next evaluate the importance of autophagy in this process, the expression of Beclin-1, a well-known regulator [26], was targeted by RNA interference. Remarkably, deprivation-induced GSC apoptosis was quelled when Beclin-1 levels were reduced (Fig. 2g). Alternatively, treatment with the lysosomotropic agent Chloroquine, a widely use autophagy inhibitor, also reduced mitogen deprivation-driven apoptosis (Fig. 2h). Taken together, our results show that GSC viability relies on the availability of mitogens, as withdrawal results in autophagy and apoptosis.

### mTor inhibition provokes autophagy and apoptosis in GSCs

**Figure 3:**
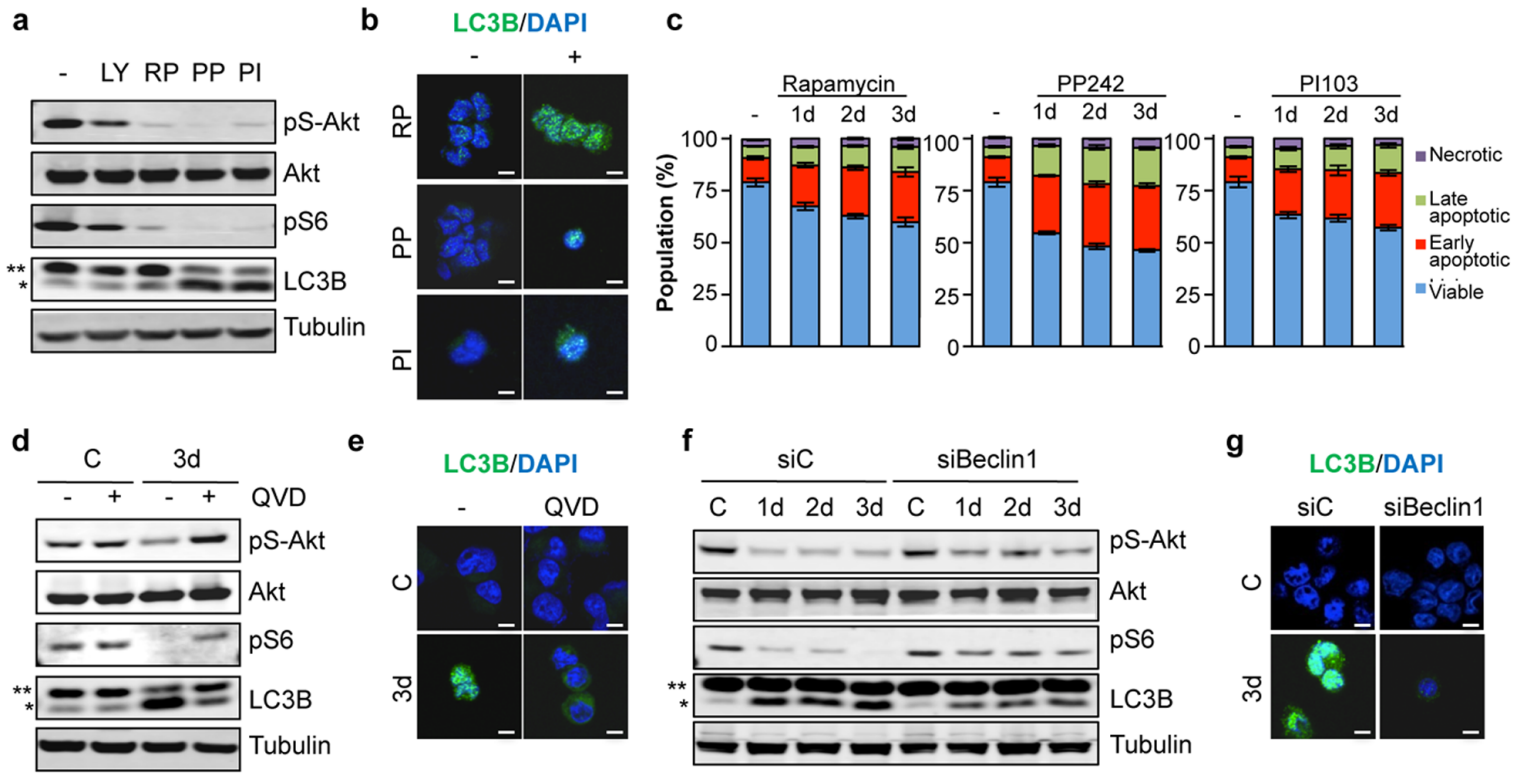
mTOR inhibition provokes autophagy and apoptosis in GSC. a-c) GSC#1 were treated with DMSO (–), LY294002 (LY, 10 μM), Rapamycin (RP, 50nM), PP242 (PP, 1 μM) and PI103 (PI, 10 μM). a) Protein lysates were analyzed after 1 day by western blot for the indicated antibodies. b) LC3B puncta were examined under confocal microscope after 3 days. Scale bars: 5 μm. c) Flow cytometry analysis of Annexin V-FITC/PI staining was performed as described in 2f). d-g) GSC#1 were pre-treated with vehicle (DMSO) or QVD (10 μM) for 45 min and mitogen-deprived for 3 days in the absence or presence of the drug (d-e). Alternatively, GSC#1 were transfected with siRNA against Beclin-1 or a control siRNA (siC) for 48 hours. Cells were mitogen-deprived as indicated and collected 5 days post-transfection (f-g). Protein extracts were analyzed by western blot for the indicated antibodies (d, f). ** non-processed form; * processed form. LC3B staining was examined by confocal microscopy (e, g). Scale bars: 5 μm. Graph represents mean+s.d. of three independent experiments. Each panel is representative of three independent experiments.

Although the mTor pathway has been linked to autophagy and cell death in the bulk of cancer cells [27], its involvement in cancer stem cell survival is unclear. In that view, mTor was pharmacologically inhibited in GSCs through four different means: LY294002, rapamycin, PP242, and PI103, which target PI3K, mTor complex 1, mTOR kinase activity, and both PI3K and mTor, respectively [11], [28]. A 24 hour-treatment of GSCs cultured in mitogen-containing medium strongly impaired mTor signaling, and led to LC3B lipidation and punctated staining (Fig. 3a-b). As a consequence, cell viability was gradually reduced (Fig. 3c). Thus, mTor activity is required to restrain GSC apoptosis and autophagy. Strikingly, inhibition of caspase-dependent apoptosis with QVD restored both the activation of the mTor pathway and low levels of LC3B lipidation under deprivation conditions (Fig. 3d-e). On the other hand, knocking down Beclin-1 modestly, but consistently, contravened mitogen withdrawal-induced mTor inhibition (Fig. 3f-g). Therefore, blockade of either autophagy or apoptosis can restore mTor pathway activation in patient-derived GSCs.

### Brain endothelial cells protect GSCs from mitogen deprivation-induced cell death

**Figure 4:**
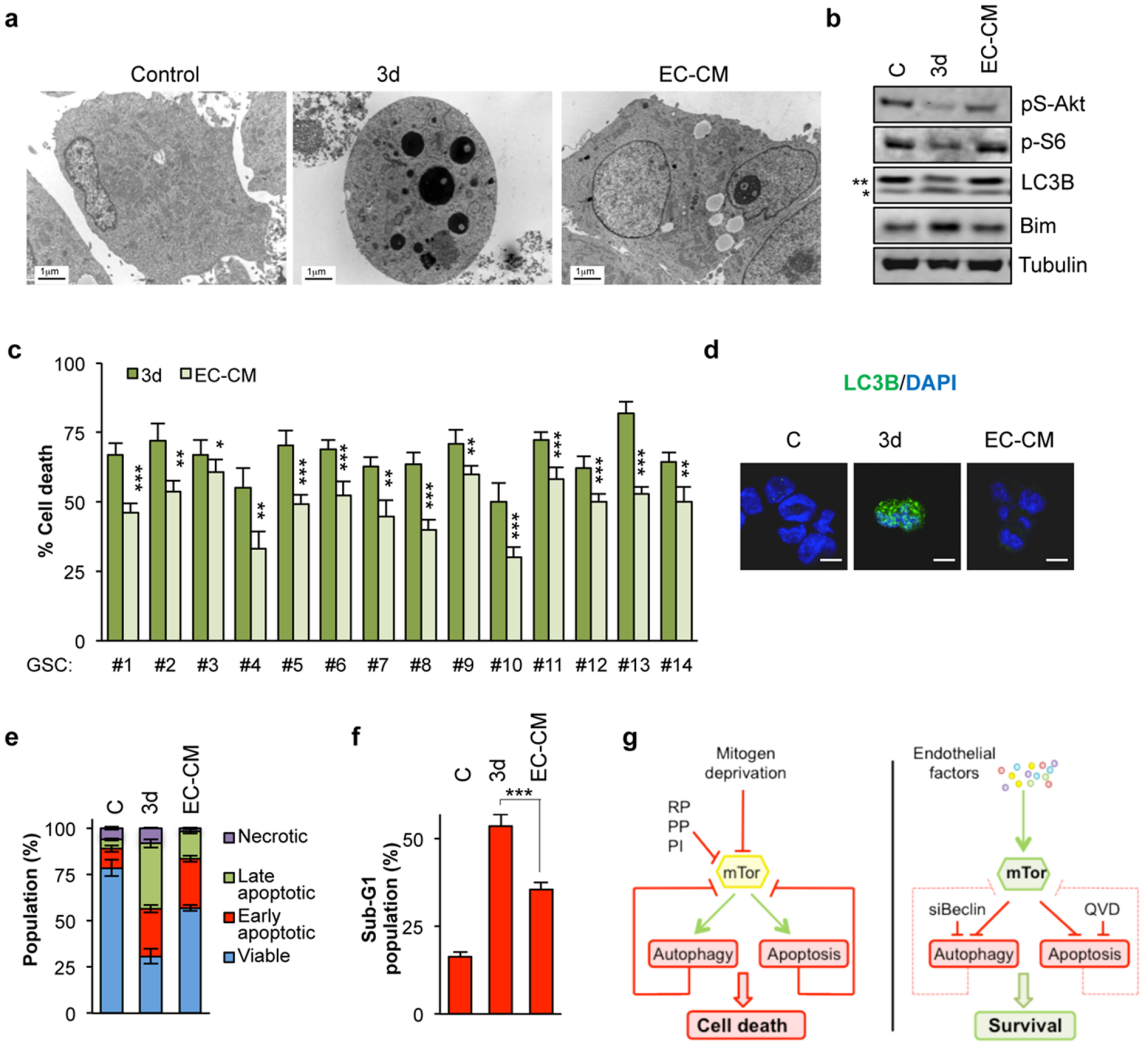
Brain endothelial cells protect GSC against mitogen deprivation-induced autophagy and apoptosis. a-d) GSC#1 were cultured for 3 days with control medium (C), deprivation medium (3d) or endothelial-derived conditioned medium (EC-CM). a) Electron microscopy analysis revealed a protective effect of EC-CM against apoptosis when compared to 3 day-starved cells. Scale bars: 1 μm. b) Protein extracts were analyzed by western blot for the indicated antibodies. ** non-processed form; * processed form. c) GSCs #1–14 were cultured for 3 days with deprivation medium or EC-CM and PI incorporation was measured by flow cytometry. Percent of cell death was represented as the mean+s.d. of three independent experiments. Student’s t-test: ^***^ *P*<0.001, ^**^ *P*<0.01, ^*^ *P*<0.05. d) LC3B puncta was examined by confocal microscopy. Scale bars: 5 μm. e-f) Annexin V-FITC/PI staining (e), as well as PI staining alone (f), were used to evaluate apoptosis as described in 1f-g. Each panel is representative of three independent experiments. g) Schematic representation of the central role of mTor in regulating mitogen deprivation-induced autophagy and apoptosis in GSCs (left panel). The positive effect of endothelial factors on this pathway is illustrated (right panel).

GSCs reside within a vascular niche, which provides a specific and confined microenvironment that is involved in the regulation of stem cell self-renewal and fate [11], [29]–[31]. Because the brain endothelial cell secretome modulates the mTor signaling nexus in GSCs [11], [18], we hypothesized that it might function as a gatekeeper against mitogen deprivation-induced cell death. To test this hypothesis, GSCs were cultured with endothelial cell conditioned medium (EC-CM) in the absence of other additives. In this condition, fewer autophagic and apoptotic features were observed by electron microscopy, when compared to deprived cells (Fig. 4a). Endothelium-derived factors also protected GSCs from deprivation-induced autophagy and apoptosis, as illustrated by the absence of lipidated and punctated LC3B, reduced Bim levels, enhanced cell viability and decreased cell death (Fig. 4b-f). Thus, endothelial-derived factors preserve mTor activation and thereby allow GSC self-renewal, in the absence of mitogen additives.

## Discussion

Cancer stem cells (CSCs) initiate tumor formation, contribute to relapse and, consequently constitute a promising cellular target to contain tumor progression. Different options can be considered to limit CSC maintenance and self-renewal, such as promoting their differentiation, reducing their interaction with blood vessels and inducing cell death [32], [33]. For instance, it has been described that anti-angiogenic therapies elicit GSC disappearance [9]. However, the molecular mechanisms involved are not fully elucidated yet. Therefore, understanding how CSCs survive and interact with their microenvironment is of great importance for the future development of specific strategies to eradicate this cell population. In the present work, we studied the behavior of GBM patient-derived stem-like cells under deprivation conditions, reminiscent of that found in glioblastoma. Using this *ex vivo* tool, we report that mitogens and endothelial-secreted factors sustain mTor activation, which in turn precludes both apoptosis and autophagy. Interestingly, apoptosis and autophagy can operate a negative feedback loop on mTor, since inhibition of either of these processes can restore mTor activation under deprivation conditions. Although the molecules involved in this loop are not identified yet, it is tempting to postulate that downstream targets of mTor could operate a positive control on the pathway. Collectively, our data support the notion that mTor centrally controls the fate of GSCs.

Electron microscopy analysis of mitogen-deprived GSCs revealed the progressive appearance of morphological features of both autophagy and apoptosis, which were further confirmed at the biochemical level. Likewise, our work illustrates how autophagy and apoptosis are orchestrated upon growth factor removal (Fig. 4g). Since Beclin-1 depletion, as well as chloroquine treatment impair both deprivation-induced autophagy and apoptosis, autophagy might be a necessary step for deprivation-induced cell death in GSCs. However, apoptosis inhibition can bypass deprivation-induced autophagy, indicating that these processes are inextricably interrelated. A potential molecular link could be the mTor pathway. Indeed, while aberrantly hyper-activated in GSCs, the efficient obstruction of the mTor pathway through well-known inhibitors not only provokes autophagy, but also induces apoptosis in a similar trend to that of mitogen deprivation. Thus, inhibition of this signaling axis might occur upstream of both processes.

More interestingly, blockade of either apoptosis or autophagy seems to be sufficient to guarantee mTor activation even in deprivation conditions. Our results thus suggest that mTor plays an instrumental role in GSC survival, reinforcing the idea that GSCs are addicted to mTor for their survival. Indeed, beyond the canonical actions of the mTor signaling nexus in mitogen deprivation-induced cell death and autophagy, we now report an unexpected retroactive feedback loop, where blocking these two events can re-initiate mTor activity. This strongly suggests that GSCs rely on the mTor pathway for their survival and that its sustained activity is *sine qua non* to maintain the GSC population. Thus, mTor signaling can control GSC viability through a two-pronged mechanism involving apoptosis and autophagy (Fig. 4g). To conclude, our results support the concept that endothelial cells could antagonize the negative reciprocal loop that suppress the mTor pathway in a hostile microenvironment, as mimicked by the absence of mitogens.

It is widely recognized that stem cell niches include a vascular structure, and are responsible for controlling local metabolic conditions, secreted protein concentrations, cell adhesion and communication to the surrounding matrix and neighboring cells [11], [29]–[31]. Importantly, GSCs have been identified in close proximity to brain endothelial cells, while targeting the tumor vasculature depletes the self-renewing GSC sub-population and impairs brain tumor growth [9]. *In vitro*, endothelial cells secrete protein factors that can sustain mTor activation [11]. In this report, we demonstrate that endothelial secreted factors act as trophogens that sustain GSC viability. This relies most likely on the ability of the endothelial secretome to secure neurosphere integrity and mTor activity. Therefore, this study strengthens the emerging concept that the brain vasculature supplies essential signals to maintain the cancer stem-like cell population in glioblastoma.

In summary, mitogen deprivation-induced cell death depends on mTor inactivation in GSCs. Likewise, pharmacological inhibition of mTor promotes cell death (Fig. 4g, left panel). Conversely, either endothelial factors or blockade of autophagy and apoptosis restores mTor signaling (Fig. 4g, right panel). Our results highlight the role of mTor as a keystone for GSC maintenance and support the concept of GSC addiction to this pathway for their survival. Although further studies are required to fully uncover the molecular mechanisms implicated in the regulation of mTor status in the tumor microenvironment, altering its activity might be an effective therapeutic strategy for future GBM treatment.
